# Novel Pomegranate-Nanoparticles Ameliorate Cisplatin-Induced Nephrotoxicity and Improves Cisplatin Anti-Cancer Efficacy in Ehrlich Carcinoma Mice Model

**DOI:** 10.3390/molecules27051605

**Published:** 2022-02-28

**Authors:** Steve Harakeh, Mohammed S. Almuhayawi, Isaac O. Akefe, Saber H. Saber, Soad K. Al Jaouni, Torki Alzughaibi, Yousef Almehmadi, Soad Shaker Ali, Dhruba J. Bharali, Shaker Mousa

**Affiliations:** 1 King Fahd Medical Research Center, King Abdulaziz University, Jeddah 22230, Saudi Arabia; taalzughaibi@kau.edu.sa; 2 Yousef Abdul Latif Jameel Scientific Chair of Prophetic Medicine Application, Faculty of Medicine, King Abdulaziz University, Jeddah 22230, Saudi Arabia; saljaouni@kau.edu.sa; 3 Department of Medical Microbiology and Parasitology, Faculty of Medicine, King Abdulaziz University, Jeddah 22230, Saudi Arabia; msalmuhayawi@kau.edu.sa; 4 Department of Physiology, Biochemistry and Pharmacology, Faculty of Veterinary Medicine, University of Jos, Jos 930003, Nigeria; i.akefe@uq.edu.au; 5 Laboratory of Molecular Cell Biology, Department of Zoology, Faculty of Science, Assiut University, Assiut 71515, Egypt; dr.saberh@gmail.com; 6 Department of Hematology/Pediatric Oncology, Faculty of Medicine, King Abdulaziz University, Jeddah 22230, Saudi Arabia; 7 Department of Medical Laboratory Sciences, Faculty of Applied Medical Sciences, King Abdulaziz University, Jeddah 22230, Saudi Arabia; 8 Rabigh Faculty of Medicine, King Abdulaziz University, Jeddah 22230, Saudi Arabia; yalmehmadi@kau.edu.sa; 9 Department of Anatomy, Faculty of Medicine, King Abdulaziz University, Jeddah 22230, Saudi Arabia; soadshaker@gmail.com; 10 The Pharmaceutical Research Institute, Albany College of Pharmacy and Health Sciences, Rensselaer, NY 12144, USA; dhruba.bharali@acphs.edu

**Keywords:** cisplatin, antioxidant, pomegranate, nephrotoxicity, nano-formulation

## Abstract

Cisplatin (CISP) is one of the most widely used anti-cancer chemotherapeutic agents with remarkable efficacy against various types of cancers. However, it has been associated with nephrotoxicity amongst other undesirable side effects. Pomegranate (PE) is a potent antioxidant and anti-inflammatory agent effective against cancer, with a superior benefit of not being associated with the common toxicities related to the use of conventional chemotherapeutic agents. However, the application of PE is limited by its reduced solubility and decreased bioavailability. We investigated the potential of a novel nanoparticle (NP) enclosing PE to enhance its solubility and improve its bioavailability, and efficacy to prevent CISP-associated nephrotoxicity in a mice model of Ehrlich solid carcinoma (ESC). All mice were grouped into four cohorts: (I) control, (II) tumor, (III) CISP, and (IV) CISP + PE-NPs. The data obtained demonstrated that PE-NPs was beneficial in potently ameliorating CISP-induced nephrotoxicity in ESC mice. PE-NPs significantly attenuated CISP-induced oxidative stress and lipid peroxidation in the kidney via improving activities of antioxidants (SOD, GSH, and CAT). Additionally, PE-NPs considerably decreased CISP-induced inflammation in the kidney by decreasing the levels of NF-kB, IL-1β, and TNF-α. Notably, PE-NPs did not assuage the antitumor efficacy of CISP as revealed by histological assessment and tumor weight data. In summary, PE-NPs may be a potent alternative anticancer therapy devoid of nephrotoxicity.

## 1. Introduction

The global population-based disease incidence data has reported a geometric rise in the occurrence of cancer, ranking among the leading causes of mortality and giving the global rise in risk factors especially in developing economies, it is expected to reach 28.4 million cases in 2040 [1]. Projections by the American Cancer Society estimates the numbers of new cancer cases be reach about 1,918,030 in 2022 [2]. Several ongoing studies investigating the use of different chemotherapeutic agents on several drug targets critical for the prevention and control of cancer. Owing to its DNA binding affinity and ability to induce the destruction of cytotoxic DNA, Cisplatin (CISP) remains a potent chemotherapeutic agent widely administered in the management of solid tumors [3]. Alongside impairing the innate cellular ability to induce apoptosis and repair [4], it also causes nephrotoxicity [5], myelosuppression, vomiting and nausea among other notable undesirable adverse effects [6]. Owing to its effect on the proximal tubule in rodents, nephrotoxicity has been implicated as the major side effect related to CISP administration [7,8]. Correspondingly, massive necrosis has been identified as the key histopathological feature observed in CISP-induced nephrotoxicity [9]. However, the mechanism of action by which CISP induces nephrotoxicity remain unclear.

Prominent among the suggested mechanisms include the generation free radicals, inflammation, apoptosis, and hypoxia. The application of complementary treatment alongside conventional therapy yields improved results and minimal resistance and undesirable effects. Consequently, in recent years, more studies have centered on the identification of cisplatin adjuvant candidates to mitigate the associated adverse effects. Although many herbal and synthetic antioxidants have been investigated for this purpose there is a paucity of information on a definite supplement that can effectively prevent CISP-induced nephrotoxicity [5]. Pomegranate (*Punica granatum* L.) belongs to the family Punicaceae, a common fruit derived from the deciduous tree of *Punica* L. genus [10,11].

Pomegranates originated from the Middle East but are now widely produced in mild-to-moderate climates including the United States of America, South Asia, and the Mediterranean. The pomegranate season is at its peak in late autumn and early winter (usually between September and November) [12,13]. Many studies on the various components obtained from pomegranate have shown no harmful effects of the dosages tested. Pomegranate’s non-toxic properties were validated in histopathological examinations on OF-1 mice. Furthermore, no side effects or unfavorable changes in urine or blood were detected in a trial of 86 overweight humans who received 1420 mg/day of pomegranate fruit extract in tablet form for 28 days [12,14]. Indeed, pomegranate has several therapeutic and pharmacological properties, which may be ascribed to the presence of numerous phytochemicals including phenolic acids, flavonoids, alkaloids phytocompounds, organic acids, gallotannins, anthocyanidins, ellagitannins, flavonones, flavonols, anthocyanins, fatty acids and lipids, and lignans have been isolated from pomegranate [10,15,16,17,18]. Anthocyanins, tannins, and catechins in pomegranate juice and peel also have a significant antioxidant activity [10,11,19,20]. Pomegranate has been used to mitigate cancer, memory impairment, and arteriosclerosis and lowering high cholesterol levels [19,20]. The efficacy of ethno-therapeutic agents in the treatment of cancer has been greatly enhanced by the recent incorporation of nanotechnology via the use of natural nanoparticle (NP) remedies which boost both bioavailability and effectiveness of potential therapeutic candidates [21,22,23]. Consequently, we sought to investigate the potential role of PE-NPs and their possible protective mechanism against CISP-induced nephrotoxicity in an Ehrlich solid carcinoma mice model.

## 2. Materials and Methods

### 2.1. Synthesis of Pomegranate-Nanoparticles (PE-NPs)

The NP-PE used in this study was prepared by double emulsion technique of encapsulating pomegranate (fruit extract) in Pluronic 127 non anionic surfactant and PVA and PLGA organic polymers. PE (40 mg), stearic acid (100 mg), lecithin (150 mg), and cholesterol (80 mg) were combined in ethanol (10 mL) and heated at 70 °C for 10 min as previously described [24,25].

### 2.2. Characterization of PE-NPs

The size distribution and zeta potential of PE-NPs in aqueous diffusion was identified via Electrophoretic Light Scattering (ELS) and Dynamic Light Scattering (DLS) methods as previously described in our previous publication on the testing of the same compound in a different biological system [26,27].

### 2.3. Assessment of the Loading Ratio (LR) and Efficiency of Encapsulation (EE)

As previously described, the EE of PE-NPs was ascertained by evaluating the ratio of the quantity of pomegranate encapsulated in the nanoparticle in comparison to the initial quantity fed, as described in Equation (1):

(1) $$\mathrm{EE}=\frac{\mathrm{Quantity}\;\mathrm{of}\;\mathrm{pomegranate}\;\mathrm{encapsulated}\;\times 100}{\mathrm{Initial}\;\mathrm{quantity}\;\mathrm{of}\;\mathrm{pomegranate}}$$

The LR was ascertained by evaluating the ratio of the quantity of pomegranate encapsulated to the total weight of entire nanoparticle formulation, as described in Equation (2):

(2) $$\mathrm{LR}=\frac{\mathrm{Quantity}\;\mathrm{of}\;\mathrm{pomegranate}\;\mathrm{encapsulated}\;\times 100}{\mathrm{Entire}\;\mathrm{weight}\;\mathrm{of}\;\mathrm{formulation}}$$

### 2.4. Animals and Treatment

Twenty-eight (28) ten-week-old adult Wistar albino male mice weighing approximately 22–30 g were used as subjects in this study. All animals were housed in the animal breeding facility of King Fahd Medical Research Centre, King Abdulaziz University (KAU), Jeddah, Saudi Arabia. All effort to minimize stress due to the handling of animals was employed and the experiment was conducted in line with the approved stipulations of the committee for ethical use and care of animals at KAU university (Number: 02-CEGMR-BIOETH-2022). All mice were acclimatized to the new environment and feeding for 2 weeks prior commencing the experimental procedure. All animals were allowed access to water and fed commercially purchased rat chow ad libitum. The CISP administered to mice was obtained from Mylan Institutional LLC, Rockford, IL, USA.

### 2.5. ESC Induction

Viable Ehrlich ascites carcinoma cells was acquired from the National Cancer Institute, Cairo, Egypt, and administered via intramuscular injection (2.6 × 10^6^ cells; 0.2 mL PBS/mouse) to induce ESC [24]. Following 10 days post implant, a palpable tumor growth was observed in all the treated mice. Thereafter, the ESC animals were grouped into 3 cohorts (*n* = 7), aside from the untreated group of control mice bearing no tumor. Oral administering of PE-NPs was carried out daily for 14 days following a single intraperitoneal dose of CISP. Phosphate buffered saline (PBS) solution with normal pH was administered orally to all the control animals. The dosage of CISP administered was in line with previous studies [25]. PE-NPs was administered at a dose of 1.47 mg/kg as previously described [26]. An outline of the experimental groups and the dosage of the different agents administered is outlined in Table 1.

### 2.6. Samples Collection and Storage

Fourteen days following the treatment, all mice were anesthetized using ether, and blood samples were collected by cardiac puncture. Thereafter, serum samples were separated by centrifuging the blood at 5000× *g* for 5 min at 4 °C. The samples were then stored at −80 °C for further processing. All the animals were then sacrificed, and tissue samples were collected and for estimation of inflammation, oxidative stress, and histopathology.

### 2.7. Assessment of Kidney Function

Serum concentrations of uric acid, creatinine, urea, C-reactive protein, and cystatin C were estimated by the aid of an ELISA kit (MyBioSource, San Diego, CA, USA, catalogue No. MBS9719084, MBS9719085, MBS2903804, MBS2600001, and MBS763996 respectively), following the manufacturers guidelines.

### 2.8. Determination of Oxidative Stress Indicators

The activities of endogenous antioxidants, superoxide dismutase (SOD), catalase (CAT), and reduced glutathione (GSH), alongside oxidative stress indicators malondialdehyde (MDA) in kidney tissues which were estimated with the aid of an ELISA kit (MyBioSource; USA, catalogue No. MBS726781, MBS268427, MBS036924, and MBS265966 respectively following the manufacturers guidelines.

### 2.9. Determination of Indicators of Inflammation

The concentrations of indicators of inflammation in kidney tissues including IL-1β, TNF-α, and NF-kB were determined using ELISA kits of MyBioSource (San Diego, CA, USA) catalogue No. MBS2507393, MBS825017, and MBS268833, correspondingly, following the guide of the manufacturer’s.

### 2.10. Histopathological Investigation

As previously described, kidney and tumor sample sections were stained using haematoxylin and eosin (H & E) and observed for histopathological variations under the microscope [27].

### 2.11. Data Analysis

Data obtained from each group was expressed as mean ± standard error of the mean (SEM; *n* = 7) and compared with other groups using one-way ANOVA followed by Tukey’s post hoc test, using GraphPad Prism version 7. Values of *p* ≤ 0.05 were considered statistically significant.

## 3. Results

### 3.1. Nutritional Composition of Pomegranate Fruit

The nutritional composition of pomegranate fruit was analyzed and represented in Table 2.

### 3.2. Preparation and Physicochemical Characterization

The nanoparticles of PE were prepared following a previously described procedure by Almuhayawi et al., 2020. The zeta potential, Z-average particle size, entrapment efficiency, and drug loading capacity were evaluated using HPLC-UV to validate the efficiency of the preparation technique to preclude loss of the active drug.

### 3.3. Effect of PE-NPs on Oxidative Stress, Antioxidant, and Inflammatory Markers in Kidney Tissues

**Figure 1:**
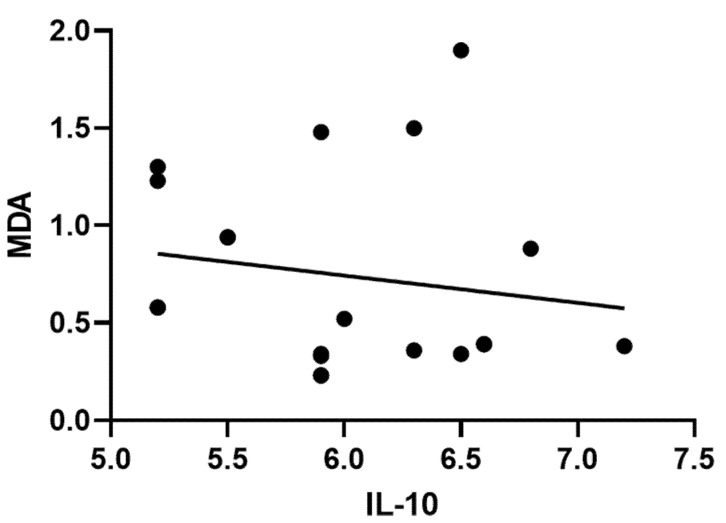
Correlation between serum MDA levels and IL-10 in following treatments.

The administration of CISP to the mice groups bearing ESC considerably decreased the activities of endogenous antioxidants in kidney tissues (SOD, GSH, and CAT) when compared to the mice in the Ehrlich tumor groups and the control group. Similarly, treatment of Ehrlich solid tumor mice with CISP significantly increased the lipid peroxidation (an index of oxidative stress) as indicated by the higher concentration of MDA in kidney tissues in comparison to the ESC and control mice. The group of mice treated with PE-NPs + CISP exhibited a significantly reduced concentration of MDA in comparison to the levels observed in the CISP group mice (Table 3). Notably, the mice group administered PE-NPs + CISP exhibited a significantly improved SOD, GSH, and CAT activities when compared those in the CISP group (Table 3, Figure 1).

### 3.4. Effect of PE-NPs on Inflammatory Markers in Serum

Administration of CISP significantly increased inflammatory markers of IL-10, TNF-α, and NF-kB in kidney tissues of mice induced with ESC in comparison to the control animals and ESC mice groups. The group of animals treated with PE-NPs + CISP showed significantly improved levels of IL-10, TNF-α, and NF-kB levels when compared with the group of animals treated with CISP (Table 3).

### 3.5. Effect of PE-NPs on Indicators of Kidney Function in Serum

**Figure 2:**
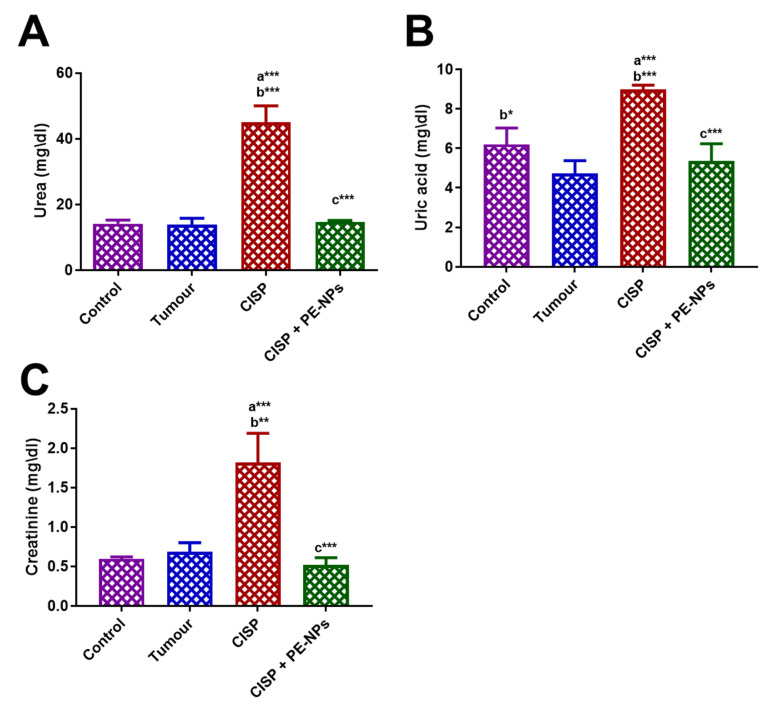
Effect of pomegranate nanoparticles (PE-NPs), on serum kidney function markers estimated following cisplatin (CISP) treatment in ESC mice. (**A**); Urea, (**B**); Uric acid; and (**C**); Creatinine. Results are expressed as mean ± SEM (*n* = 5). ^a^ Significant difference against the control group. ^b^ Significant difference against Ehrlich tumor group. ^c^ Significant difference against CISP group. *** *p* ≤ 0.001, ** *p* ≤ 0.01, and * *p* ≤ 0.05.

**Figure 3:**
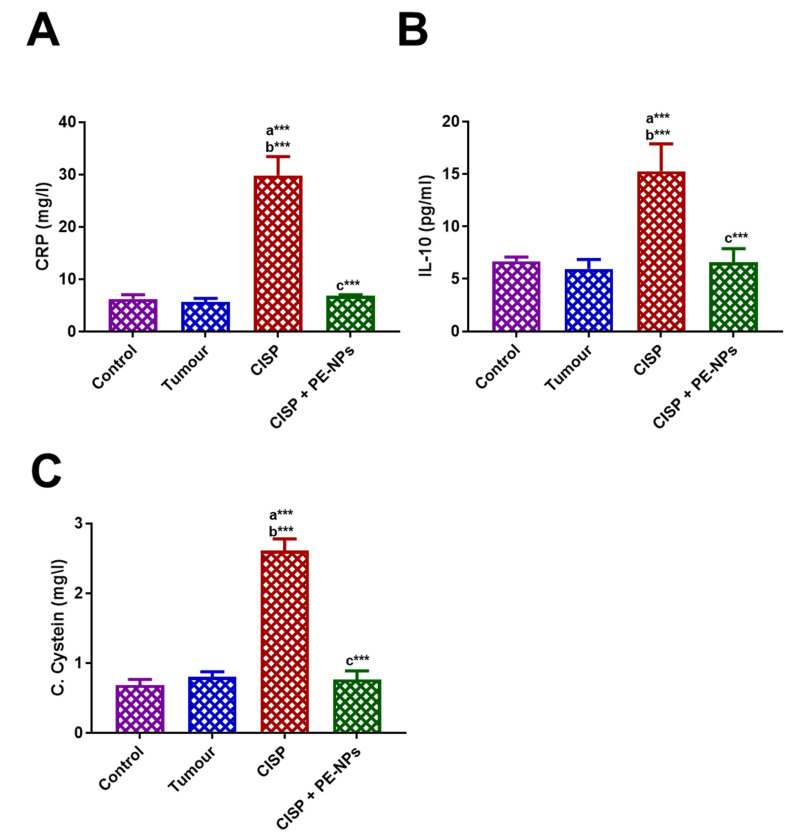
Effect of PE-NPs on serum inflammatory markers. (**A**) CRP, (**B**) IL-10, and (**C**) C-Cysteine estimated following cisplatin (CISP) treatment in ESC mice. Results are expressed as mean ± SEM (*n* = 5). ^a^ Significant difference against the control group. ^b^ Significant difference against Ehrlich tumor group. ^c^ Significant difference against CISP group. *** *p* ≤ 0.001.

Serum concentrations of urea, uric acid, creatinine, C- reactive protein, and cystatin C. The indicators of kidney function (serum levels of uric acid, urea, and creatinine) increased significantly in ESC mice treated with CISP when compared to mice in either control or ESC groups (Figure 2). All animals administered PE-NPs + CISP exhibited improved serum kidney functions as significantly reduced serum creatinine, uric acid, cystatin C, and urea was recorded when compared to the group of mice treated with CISP alone (Figure 2). Besides, administration of PE-NPs improved the levels of serum inflammatory markers including CRP, IL-10, and C-Cysteine estimated following cisplatin (CISP) treatment in ESC mice (Figure 3).

### 3.6. Effect PE-NPs on the Anticancer Activity of CISP in ESC Mice

**Figure 4:**
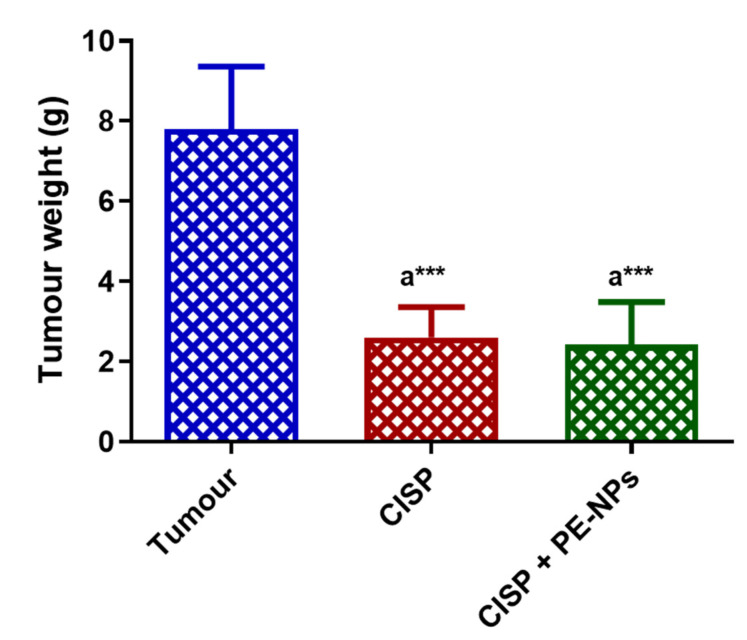
Effect of combination of CISP with PE-NPs on ESC tumor weight. Results are expressed as mean ± SEM (*n* = 5). ^a^ Significant difference against Ehrlich tumor group. *** *p* ≤ 0.001.

The administration of CISP to mice bearing ESC considerably reduced the weight of the tumor when compared to the ESC group of animals that were untreated. Notably, the group of animals bearing ESC treated with PE-NPs + CISP also showed significantly reduced tumor weight in comparison with the untreated ESC group of mice. However, no significant variation in the weight of tumors was recorded between the ESC bearing mice treated with CISP only and those administered a combination of PE-NPs + CISP (Figure 4).

### 3.7. Effect of Combination of CISP with PE-NPs on Kidney Histopathology in ESC Mice

**Figure 5:**
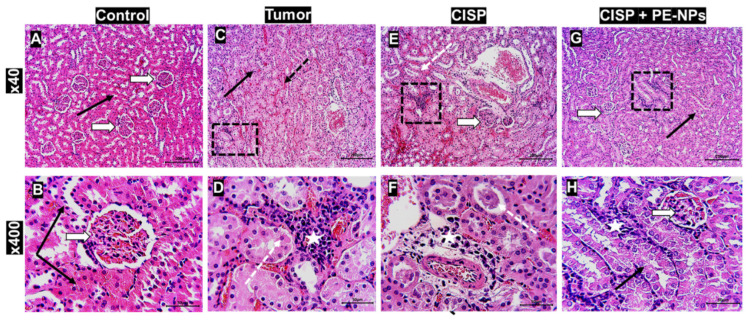

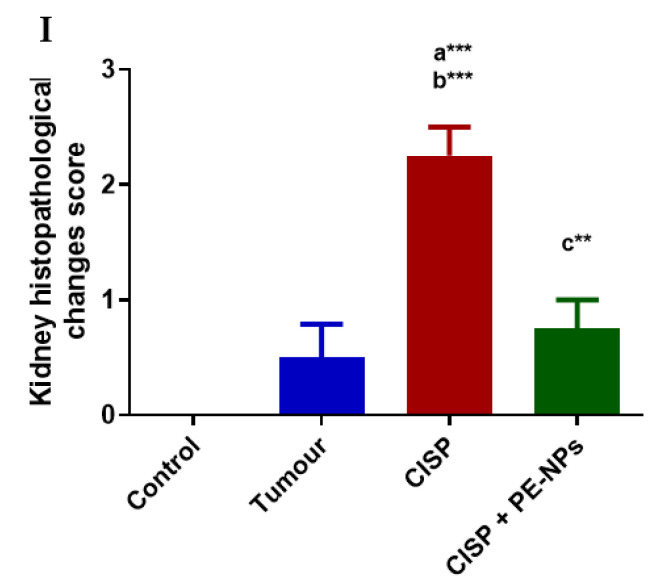
Effect of the combination of CISP with PE-NPs on kidney tissue histopathology in ESC mice model. Kidney sections were stained by H & E and photographed at low power × 40 & high-power × 400 to show: Control (**A**,**B**): showing normal kidney parenchyma; renal corpuscle and glomeruli (white arrow) and kidney tubules (black arrows). Tumor (**C**,**D**): showing massive infiltration by neoplastic viable cells among the tubules with capillary congestion (dotted black arrows) and unstained mild basal degeneration of tubule cells (black arrows). CISP (**E**,**F**): showing marked vascular congestion (dotted arrows), dilatation of tubular lumen with cast formation (black arrows) with decreasing degenerated neoplastic cells (dotted square and white star) tubules showed dark nuclei (apoptosis) and luminal casts (black arrows). Tumor + CISP + PE – NPs (**G**,**H**): showing preservation of kidney tubule structure and degeneration of neoplastic cells (dark nuclei; dotted square and star). (**I**) The bar plot shows the scoring for the histopathological changes in the kidney as observed in the different sections. Results are expressed as mean ± SEM (*n* = 5). ^a^ Significant difference against the control group. ^b^ Significant difference against Ehrlich tumor group. ^c^ Significant difference against CISP group. *** *p* ≤ 0.001 and ** *p* ≤ 0.01.

The administration of CISP to mice bearing ESC caused the permeated neoplastic cells in the tissue sections to look degenerated and less viable. In addition, CISP induced considerable distortion of the kidney parenchyma with deformation and atrophy of the glomeruli and renal corpuscle. However, it caused marked degeneration of the epithelium lining the kidney tubule (distortion of small nuclei stainless cytoplasm, and inflamed cells). Conversely, the group of animals treated with the combination of PE-NPs + CISP showed considerable preservation of structures in the parenchyma of the kidney with minor distortion of the renal corpuscles. The epithelial lining of the kidney tubules appeared healed such as that seen in the sections from control mice (Figure 5).

### 3.8. Evaluation of the Expression of Caspase-3 Immuno in Kidney Tissue Treated with the Combination of CISP and PE-NPs in ESC Mice

**Figure 6:**
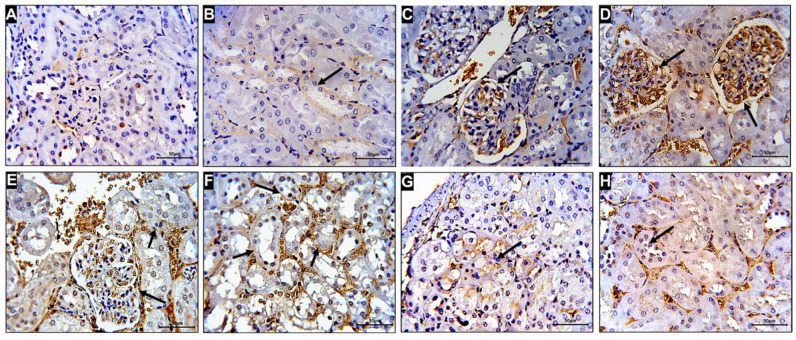
Section from cortex and medulla of mice kidney immuno-stained for caspase-3 (marker of apoptosis). (**A**,**B**), showing normal control with mild positive immuno-staining in renal corpuscle glomerular capillaries (white arrow) and peritubular medullary vessels (black arrow). (**C**,**D**) showing moderate increase in caspse-3 immunostaining in glomerular capillaries and peritubular cortical and medullary vessels in tumor mice group (black arrows). (**E**,**F**) showing marked increase in caspas3-immunostaining in glomerular capillaries and peritubular cortical and medullary vessels and basal parts of dilated distal tubule cells of cisplatin treated mice (black arrows). (**G**,**H**), showing potential decrease in caspse-3 immuno-expression in tumor group mice treated with cisplatin and nanoparticles of pomegranate.

Figure 6 showed a mild increase in the immuno-expression of caspase-3 in both affected glomerular capillaries and medullary peritubular vessels of the kidney tissues of the control mice. In contrast, kidney tissues from untreated ESC mice showed marked elevation in the expression of caspase 3. Conversely, the ESC mice group treated with CIS + NPs pomegranate showed marked reduction in the immuno-expression of caspase-3 with well-preserved renal histoarchitecture.

### 3.9. Effect of Combination of CISP with PE-NPs on Histopathological Features in ESC Mice

**Figure 7:**
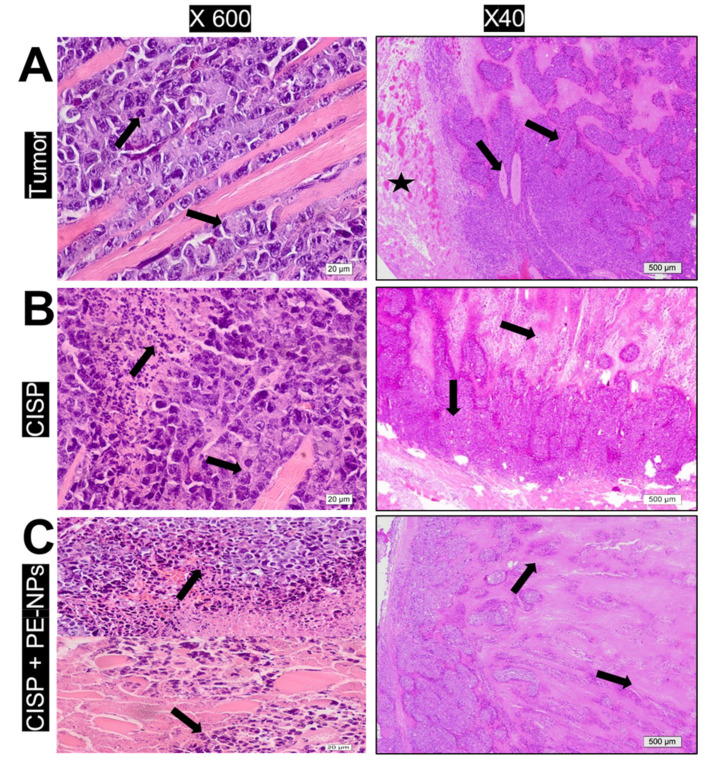
Effect of combination of CISP with PE-NPs on ESC histopathology. Sections are H & E-stained low power (right side × 40) and high power (left side × 600). (**A**) Tumor sections showing well-vascularized bed. Note the massively proliferating neoplastic cells infiltrating the muscles which have pleomorphic vesicular active nuclei with numerous mitotic figures (arrows). (**B**) CISP sections show reduced tumor vascularity (star). Neoplastic cells markedly reduced in density with wide regions of tumor necrosis (pink stained area) and small degenerated pyknotic nuclei (arrows). (**C**) CISP + PE-NPs sections showing reduced proliferation in cell density. Wider areas of tumor necrosis and degeneration of neoplastic cells is evident with fragmented darkly stained nuclei (arrows).

The administration of CISP to mice bearing ESC induced a considerable reduction in the tumor weight when contrasted with the Ehrlich tumor mice group. The administering of the combination of CISP with PE-NPs in ESC mice yielded a significant reduction in the tumor weight compared to ESC mice group. No statistically significant variation in tumor weight was noted between the mice treated with only CISP and those administered with the combination of CISP + PE-NPs (Figure 7).

Figure 7 shows that, compared to marked tumor vascularity and high density of viable neoplastic cells, large cells with pleomorphic nuclei with dominant abnormal mitotic features (arrows) appear in Ehrlich solid tumor. Tumor tissue showed large acidophilic regions that represent necrotic tissue while significant tumor regression was evidenced in the group treated with combination of CISP + PE − NPs. The histopathological observation of tumor sections from the different experimental animal groups stained with H & E further confirms this finding. The section of the tumor showed a significant increased thickening of the vascular bed alongside massive proliferating neoplastic cells, cell infiltration with pleomorphic vesicular active nuclei and numerous mitotic figures. Treatment with CISP caused a significant reduction in both tumor neoplastic cell density and vascularity. In tissue sections from CISP treated ESC mice, there were wide regions of small degenerated pyknotic and tumor necrosis. The combination of PE-NPs with CISP yielded a greater reduction in the proliferation and cell density, degeneration of neoplastic cells, wider regions of tumor necrosis, and uneven darkly stained nuclei (Figure 7). 

## 4. Discussion

Pomegranate is a derivative of *Punica* L., and possesses antioxidant properties; however, its low aqueous solubility and bioavailability limits its effectiveness [28]. The bioavailability of therapeutic agents both in vivo and in vitro is vital for its optimum effectiveness [29]. In this study, in a bid to enhance the solubility of PE, we formulated PE-NPs and characterized its properties. Accordingly, a minimal dose of PE-NPs (3 mg/kg/day) was administered orally to mice in this study. We aimed to imitate what typically ensues in cancer disease patients. Several studies have examined the potential of cancer chemotherapy toxicity in healthy animals (i.e., without cancer). However, what happens is the development of concomitant adverse effects of chemotherapy on cancer patients undergoing treatment using these drugs. Consequently, in this study we explored the potential protective effect of PE-NPs on nephrotoxicity associated with CISP in mice bearing ESC. Our result demonstrated that PE-NPs were efficient in preventing CISP-induced nephrotoxicity in ESC mice. This was corroborated by the improved kidney and liver function indicators alongside a reduction in kidney histopathology noticed upon treatment with PE-NPs in the present study. In kidney tissues, PE-NPs significantly attenuated CISP-induced oxidative stress by improving the activities of endogenous antioxidant enzymes (CAT, SOD, and GSH). PE-NPs also considerably lowered inflammation induced by CISP in kidney as evidenced by a reduction in levels of NF-kB, TNF-α, and IL-1β. This is the first report demonstrating the beneficial potential of PE-NP formulation against CISP in ESC mice model. Additionally, this result demonstrated that the nanoparticles of PE did not assuage the in vivo anticancer potential of CISP as indicated by the tumor weight and the histology result. Comprehensive evidence also validates the vital role of increased oxidative stress as the cause of nephrotoxicity induced by CISP [30,31]. Moreover, the extent of the severity of the nephrotoxicity induced by CISP has been linked with significant rise in the levels of IL-6 and TNF-α [32,33]. TNF-α is reported to be the most essential cytokine elevated during CISP-induced toxicity and consequently, its inhibition protects against the toxic activity of CISP [34]. Similarly, a study also recently showed the beneficial effect of PE against tramadol-induced testicular toxicity via reducing oxidative stress and stimulating antioxidants [35,36,37]. Several studies including that carried out by Al-olayan et al. also demonstrated the in vivo beneficial effects of PE (*Punica granatum*) juice on male infertility induced by carbon tetrachloride in a rodent model [20,38,39]. Benzer et al. (2011) also showed that pomegranate seed extract mitigated free radical damage and enhanced the antioxidant activity under cisplatin-induced oxidative stress conditions in rabbits [40,41]. In addition, pomegranate juice extract has been shown to decrease cisplatin toxicity on peripheral blood mononuclear cells [37].

Although few studies have reported that PE protects against CISP-induced toxicity [19,42], a number of other studies have shown that PE offers no protection against CISP-induced nephrotoxicity [5,14,21,32], and this may be due to the poor solubility. Hence, in this study, PE-NPs formulation was observed to improve its efficacy by enhancing the solubility. Consequently, the data from the present study suggest that the nano-formulation PE may be a beneficial adjunct nano-nutraceutical in improving cisplatin anticancer potential to mitigate the attendant nephro-pathology.

## 5. Conclusions

In summary, the data obtained from this study indicated that the combination of PE-NPs and CISP prevented against CISP-induced nephrotoxicity by enhancing the activities of endogenous antioxidants and improving the anti-inflammatory potential of PE. Therefore, it is conceivable that PE-NPs may be a complimentary therapy to protect against CISP-induced nephrotoxicity during the treatment of cancer.

## Figures and Tables

**Table 1:** Experimental groups and the agents administered.

Groups (*n* = 7 Each)	Treatment
Group I (control)	PBS (0.2 mL)/mouse
Group II (negative control)	Tumour (2.6 × 10^6^ cells in PBS)
Group III	Tumour + CISP (3.5 mg/kg)
Group IV	Tumour + CISP + PE-NPs (1.47 mg/kg)

**Table 2:** Nutritional composition of pomegranate fruit.

Nutrients	Value Per 100 g	Units
Potassium	236	mg
Sodium	3	mg
Ascorbic acid, total	10.2	mg
Choline, total	7.6	mg
Calcium	10	mg
Iron	0.3	mg
Magnesium	12	mg
Phosphorus	36	mg
Ash	0.53	g
Carbohydrates	18.7	g
Fiber	4	g
Sugars, total	13.67	g
Water	77.93	g
Energy	83	Kcal
Protein	1.67	g
Total lipid (fat)	1.17	g

**Table 3:** Effect of pomegranate nanoparticles (PE-NPs), on activity of antioxidant enzymes, oxidative stress (MDA), and inflammatory markers in kidney tissues measured during treatment with cisplatin (CISP) in Ehrlich solid carcinoma mice model.

	GSH (ng/mL)	SOD (u/mL)	CAT (Mu/L)	MDA (nmol/mL)	TNF-α (pg/mL)	IL10 (pg/mL)	NFKβ (ng/mL)
Control	12.86 ± 0.51	158 ± 2.68	111.2 ± 4.2	0.316 ± 0.036	11.34 ± 0.32	6.18 ± 0.17	14.04 ± 0.61
Tumour	17.1 ± 0.84	176.6 ± 4.12	117.8 ± 1.91	0.438 ± 0.06	12.22 ± 0.372	5.82 ± 0.27	16.18 ± 0.79
CISP	3.6 ± 0.36 ^ab^	82.2 ± 2.44 ^ab^	55 ± 4.21 ^ab^	1.482 ± 0.12 ^ab^	23.28 ± 1.44 ^ab^	13.68 ± 0.36 ^ab^	64.06 ± 4.05 ^ab^
CISP + PE-NPs	17.84 ± 1.25 ^ac^	183 ± 7.2 ^ac^	119.6 ± 1.12 ^c^	0.732 ± 0.12 ^ac^	12.9 ± 0.46 ^c^	6.2 ± 0.35 ^c^	14.46 ± 0.71 ^c^
*p* value	<0.0001	<0.0001	<0.0001	<0.0001	<0.0001	<0.0001	<0.0001

Data are expressed as mean ± SEM (*n* = 5). ^a^ = significantly different from the value in the control group (*p* < 0.05). ^b^ = significantly different from the value in the tumour group (*p* < 0.05). ^c^ = significantly different from the value in the CISP group (*p* < 0.05).
